# Geographic Origin Discrimination of Millet Using Vis-NIR Spectroscopy Combined with Machine Learning Techniques

**DOI:** 10.3390/foods10112767

**Published:** 2021-11-11

**Authors:** Muhammad Hilal Kabir, Mahamed Lamine Guindo, Rongqin Chen, Fei Liu

**Affiliations:** 1 College of Biosystems Engineering and Food Science, Zhejiang University, 866 Yuhangtang Road, Hangzhou 310058, China; mhkabir@atbu.edu.ng (M.H.K.); guindo@zju.edu.cn (M.L.G.); chenrq@zju.edu.cn (R.C.); 2 Department of Agricultural and Bioresource Engineering, Abubakar Tafawa Balewa University, Bauchi PMB 0248, Nigeria; 3 Key Laboratory of Spectroscopy Sensing, Ministry of Agriculture and Rural Affairs, Hangzhou 310058, China

**Keywords:** millet, near-infrared spectroscopy, geographic origin, machine learning

## Abstract

Millet is a primary food for people living in the dry and semi-dry regions and is dispersed within most parts of Europe, Africa, and Asian countries. As part of the European Union (EU) efforts to establish food originality, there is a global need to create Protected Geographical Indication (PGI) and Protected Designation of Origin (PDO) of crops and agricultural products to ensure the integrity of the food supply. In the present work, Visible and Near-Infrared Spectroscopy (Vis-NIR) combined with machine learning techniques was used to discriminate 16 millet varieties (*n* = 480) originating from various regions of China. Five different machine learning algorithms, namely, K-nearest neighbor (K-NN), Linear discriminant analysis (LDA), Logistic regression (LR), Random Forest (RF), and Support vector machine (SVM), were used to train the NIR spectra of these millet samples and to assess their discrimination performance. Visible cluster trends were obtained from the Principal Component Analysis (PCA) of the spectral data. Cross-validation was used to optimize the performance of the models. Overall, the F-Score values were as follows: SVM with 99.5%, accompanied by RF with 99.5%, LDA with 99.5%, K-NN with 99.1%, and LR with 98.8%. Both the linear and non-linear algorithms yielded positive results, but the non-linear models appear slightly better. The study revealed that applying Vis-NIR spectroscopy assisted by machine learning technique can be an essential tool for tracing the origins of millet, contributing to a safe authentication method in a quick, relatively cheap, and non-destructive way.

## 1. Introduction

In most countries of Asia and Africa, millet is a significant crop. For centuries, it has been considered the staple food for many people living in dry or semi-dry areas of Asia and Africa. Nearly 10,000 years ago, millet was cultivated in East Asia [1]. It can be grown in poor fertile soils and is drought-tolerant [2,3]. Many developing countries in Africa and Asia consume millets as a primary food and produce traditional alcoholic and non-alcoholic beverages, particularly in India, China, and Eastern and Southern Europe. During periods of famine, it is the primary food crop of choice. The cultivation of millets primarily focuses on forage consumption in countries such as the US, Argentina, Brazil, Australia, and South Africa [4]. The quality of food consumed worldwide is becoming an issue of growing concern among consumers, resulting in a worldwide need to establish traceability, authenticity, and originality of agricultural products. The food traceability system, which the European Union officially recognized (E.U.) in 2000, is an essential tool for protecting consumers against contamination [5]. Keeping foods free of contamination and illustrating the complete identification of products receives significant attention in many countries. Given the above, it has become evident that a precise way for geographical origin identification is necessary for food and agricultural products. Near-Infrared Spectroscopy (NIRS) is a simple, rapid, and non-destructive method that requires very little sample preparation. Vis-NIR spectroscopy, combined with machine learning algorithms, is being widely used as a reliable and successful scientific instrument in a variety of fields, including agricultural food [6], insect-based food [7], organic-fertilizers [8], whey protein powder [9], petrochemical [10], pharmaceutical [11], environment [12], metabolomics profiling [13], as well as several reviews on recent applications such as [14,15,16,17], etc. NIRS has already been established in the 1960s for cereal analyses [18]. Geographical origins of many kinds of cereal and other agricultural products have been determined using NIR in recent years, such as maize [19], walnuts [20], durum wheat [21], rice [22,23,24], turmeric [25], kudzu powder [26], Prunus Dulcis [27], Trichosanthis Fructus [28], Chinese mitten crab [29], edible oils [30], Wolfiporia cocos [31], Argentinean lemon juices [32], honey [33,34], etc.

In addition, NIRS has been applied to millets for feature extraction and variety identification [35,36] and determination of chemical properties such as moisture, proteins, and fats, etc. [37,38]. However, there are few reports on different machine learning algorithms in millet geographic origin discrimination using Vis-NIR Spectroscopy. Considering the significance of millet as a staple food for many countries worldwide, Vis-NIR Spectroscopy is hypothesized to be applied for its effective geographic origin discrimination capability.

Therefore, this study aimed to use Vis-NIR Spectroscopy combined with machine learning algorithms to discriminate 16 distinct millet species originating from different regions of China. The varietal discrimination could be beneficial for food safety and adulteration detection.

## 2. Materials and Methods

### 2.1. Sample Preparation

Tests were conducted on millet varieties commonly cultivated in different geographical regions of China and widely consumed by most people. All samples were purchased in the 2019 harvest season. Sixteen varieties (Table 1), 30 samples each, 480 samples were used.

### 2.2. Spectral Measurement

Sampling range distance (broad Frequency) was 1.5 nm, having from 350 to 2500 nm spectrum of experiment, inspection duration was 30 times, and the resolution was 3.5 nm. A fieldspec3 spectrometer collected the spectrum data test. Software A.S.D. View SpecPro (Version 6.20 Malvern Pan Analytica Ltd., Malvern, UK) exported the original results [35].

### 2.3. Data Analysis

Multivariate NIR spectra statistics (file imported in MATLAB format) were done using Unscrambler X, v10.1 (CAMO Software AS, Oslo, Norway, 2011). Principal Component Analysis (PCA) was done to analyze the data and identify millet clustering chances (similarities and differences). The graphical representations of Unscrambler X software were used to assess the presence of outliers. Before applying and validating machine learning models, the data were pretreated using six spectra preprocessing techniques which include: Multiplicative Scatter Correction (MSC), Detrend Correction (DC), Mean Centering (MC), Standard Normal Variate (SNV), First-Order Derivative (1st Der), and Second-Order Derivative (2nd Der). The Pretreated data were then employed to classify millet samples as per their respective certified origins by five different classification models, namely k-Nearest Neighbor (k-NN), Linear Discriminant Analysis (LDA), Logistic Regression (LR), Random Forest (RF), and Support Vector Machine (SVM) [28,36,37].

### 2.4. Chemometrics Study

#### 2.4.1. K-Nearest Neighbor (K-NN)

K-NN categorizes by measuring the Euclidean or Manhattan distance between distinct features [38]. Using many neighbors (k), similar features belong to a particular category. The optimized prediction ability was obtained by comparing many k values, which yielded maximum classification accuracy.

#### 2.4.2. Linear Discriminant Analysis (LDA)

LDA is a technique applied in statistics, machine learning, and pattern recognition to get a direct mixture of characteristics that describes or distinguishes many groups of things [39] and is sometimes called Fisher Linear Discriminant (FLD). A high-dimensional data sample is processed into optimal low dimension data to compress the feature section’s dimension and obtain group data. After processing, the new subsection’s data sample must minimize variation inside group $S_{w}$ (Equation (1)) and increase inter-group interval $S_{B}$ (Equation (2)). Then, the data sample may obtain the optimum distinction in the subspace of processing:

(1) $$S_{w}=\sum_{i=1}^{n}\sum_{x\in C_{i}}(x-m_{i}){\left(x_{j}-m_{i}\right)}^{T},\;$$

(2) $$S_{B}=\overset{n}{\underset{i=1}{\sum }}n_{k}\left(m_{i}-m\right){\left(m_{i}-m\right)}^{T},$$

$n_{k}$ is the number of training objects for each class, *m* is the means for each category, *m_i_* is the real mean vector, *x* is each class object, and *n* is the number of types. Fisher criteria (Equation (3)):

(3) $$J(W_{opt})\;\;\frac{W^{T}S_{B}W}{W^{T}S_{W}W}\;$$

#### 2.4.3. Logistic Regression (LR)

LR is applied to get uneven correlation in the presence of many descriptive variables. The method is entirely similar to multiple linear regression, excluding the response variable is binomial. The outcome is the effect of each variable on the uneven correlation of the noticed result of importance. The primary benefit is to circumvent contradicting influence by examining the relationship of all variables simultaneously. LR operates very much like linear regression but with a binomial response variable [40]. Logistic regression will model the chance of an outcome based on individual characteristics. Because chance is a ratio, what is be modeled is the logarithm of the chance given by:

(4) $$\log\left(\frac{\pi }{1-\pi }\right)=\beta_{1}+\beta_{1}x_{1}+\beta_{2}x_{2}+\ldots \beta_{m}x_{m}$$

where *π* indicates the probability of an event and *β_i_* is the regression coefficients associated with the reference group and the *x_i_* explanatory variables.

#### 2.4.4. Random Forest (RF)

RF uses a resolution forest, which is produced from many decision trees. The mixture of sacking (bootstrap combination) program and program organization builds a resolution structure, which utilizes several resolution diagrams as an outcome. It may also be applied for categorization and regression [41]. RF categorizer uses the sacking of resolution tree technique to bring an extensive group of trees to enhance efficiency. Assessed with other groups alike, RF needs minor hyperparameter tuning. Original sacking resolution tree gives tree-interdependence, which hurts from the effect of enormous variation. Thus, RF provides a variation minimization by establishing more uncertainty in the tree-generation technique [42].

#### 2.4.5. Support Vector Machine (SVM)

SVM algorithm is a data mining approach for classification and regression [43], which depends on the structural risk reduction principle and can overcome over-fitting problems. SVM [44] is a data evaluation technique based on machine learning, and it is commonly used in categorization [45].

## 3. Results

### 3.1. Spectra Analysis

**Figure 1:**
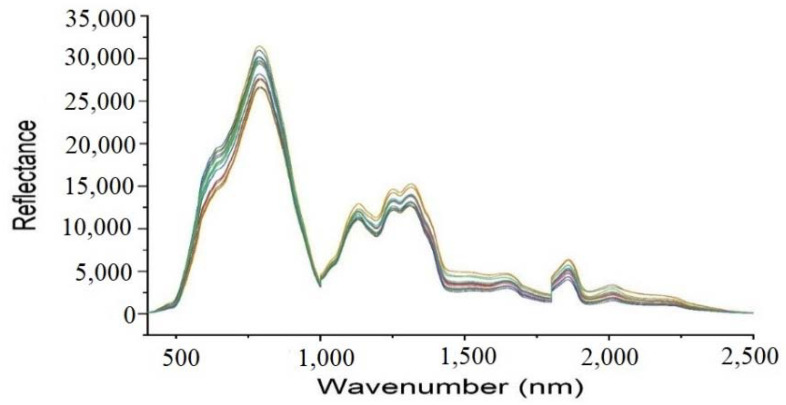
Millet Raw spectra from different geographic regions of China.

A comparison of raw spectra from different areas of China revealed no significant differences. Due to the high overlap in the raw spectrum, it was difficult to detect the distinct bands. It is also possible that the model effectiveness could be adversely affected due to the presence of noise and background information in the raw spectrum. As a means of achieving a good model and reducing background noise, spectra preprocessing is essential [28,36,37]. Six spectra preprocessing techniques, which include Multiplicative Scatter Correction (MSC), Detrend Correction (DC), Mean Centering (MC), Standard Normal Variate (SNV), First-Order Derivative (1st Der), and Second-Order Derivative (2nd Der), were comparatively applied in each model to improve the accuracy. Figure 1 presents raw spectrums of samples from different geographical regions of China.

### 3.2. Principal Component Analysis

**Figure 2:**
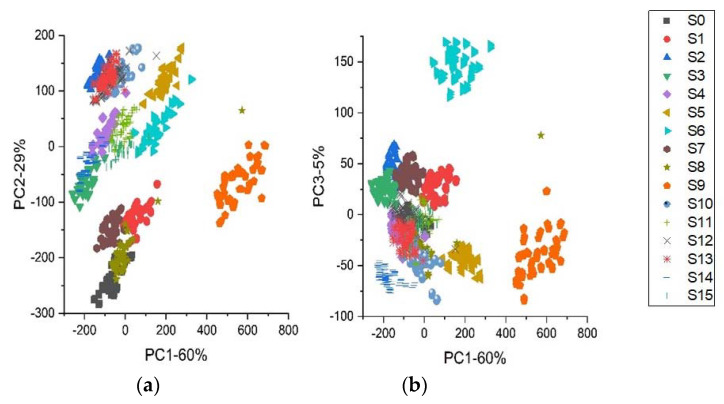
Principal Components (PCs) distribution diagrams of individual samples. (**a**) PC1-PC2; (**b**) PC1-PC3.

Principal Component Analysis (PCA) is a widely used technique in the analysis of spectral data. PCA transformed linear combinations of the original variables into new variables called principal components (PCs). These PCs are orthogonal and are positioned based on an interpretation of the variation. The first (PC) analyzed nearly all of the variations and accompanied the second, third, etc. Generally, the bulk of the variations were analyzed by the first few PCs [46]. Score biplots are usually used to depict how sample groups performed according to the two divergent PC models. Figure 2 is a plot of PCs score for each variety; a different color represents each for better visualization. They were using the first three normalization scores of PCs. As shown in Figure 2, PC1, PC2, and PC3 accounted for 60%, 29%, and 5% of the total variation, respectively.

**Figure 3:**
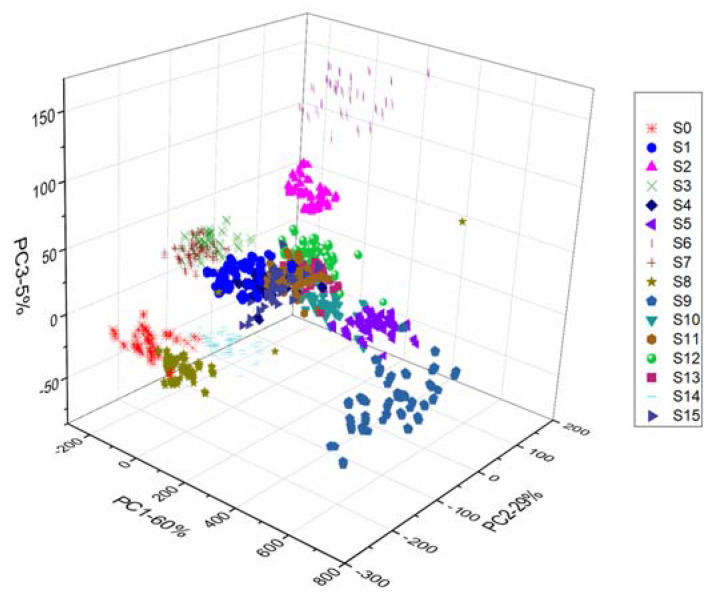
3-dimensional distribution of individual samples.

In contrast, the first three PCs accounted for 94% of the variance. It appears that the three peak PCs contain almost the complete spectral information of the different regions in the NIR. It can be seen from Figure 3 that sixteen varieties could be distinguished with little overlap. An apparent disconnection exists between the varieties. The millet samples were roughly separated based on geographical origin. It was evident from the PCA evaluation that chemical compositions differ between the different varieties. Even though the analysis displayed the cluster trend in three dimensions, the samples could not be effectively distinguished. Thus, this study employed a sufficient number of multivariate classification algorithms [28,34]. Therefore, machine learning algorithms may be an appropriate means of efficiently taking the precise development of spectral characteristics wavelengths and identifying any spectral sequence that is not identifiable by standard NIR spectrum analysis, creating feasibility for discrimination of NIR spectroscopic data.

### 3.3. Models Optimization

The following machine learning algorithms were applied to classify millet according to their geographic origin: K-NN, LDA, LR, RF, and SVM. The highest accuracy rate was selected using the cross-validation (CV) approach [28,47]. A general definition of machine learning is a program that removes the unknown features from vast quantities of information and utilizes them for estimation or tagging. As an analytical technique, it is useful primarily in finding a relationship between sample data as input and output. The method is typically applied to emerging areas, particularly for identifying the authenticity of the food. It has a strong ability in the area of food origin tracing [47]. For the geographical origin of cereals and agricultural commodities, there are several ways, depending on the analytical methods used and the statistical methods employed, which have been proposed.

#### 3.3.1. Discrimination Results from the Different Models

Data obtained from the Vis-NIR spectroscopic measurements were analyzed using machine learning algorithms. A calibration set (70%) and a testing set (30%) were used to evaluate the models and test their efficacy in classification. Cross-validation (CV) is a powerful technique to reduce overfitting likeliness [37]. ML discrimination models have both strengths and weaknesses. A small number of the ones applied in the present study demonstrated their ability to distinguish millet that originates from distinct geographic regions in China. The discrimination results are shown in Table 2.

**Figure 4:**
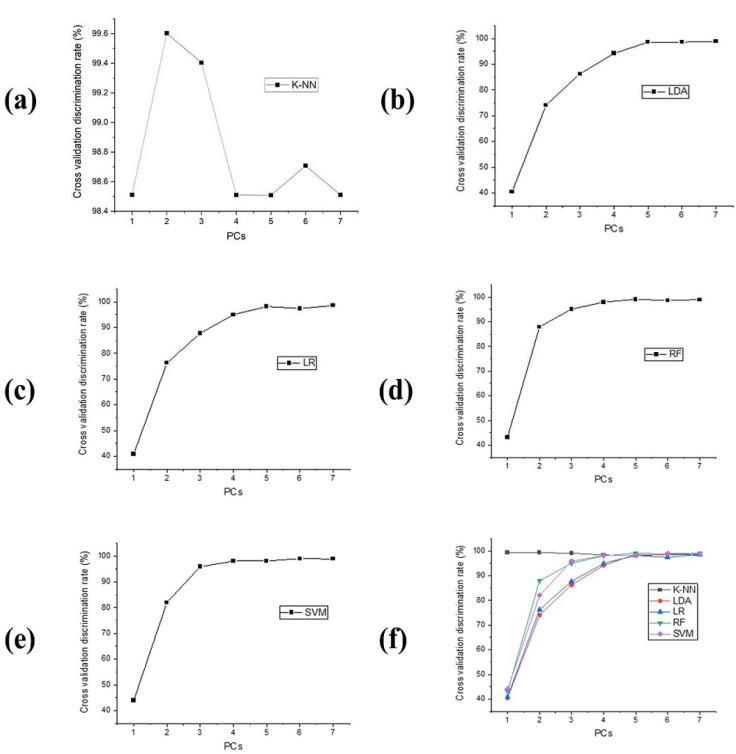
(**a**) Cross-validation discrimination rates of K-NN model at different PCs, (**b**) Cross-validation discrimination rates of LDA model at different PCs at different PCs, (**c**) Cross-validation discrimination rates of LR model at different PCs, (**d**) Cross-validation discrimination rates of RF model at different PCs, (**e**) Cross-validation discrimination rates of SVM model at different PCs, (**f**) Cross-validation discrimination rates of the five models at different PCs.

K-NN model. Figure 4a. Shows the performance of the K-NN model. K-NN is a linear and non-parametric technique that attained the best discrimination accuracy at an optimized PC = 6 and K = 2. The mean centering (MC) preprocessing procedure was found to be most excellent. As shown in Table 2, the optimal discrimination rate by the K-NN model was 99.90% for the calibrations and 99.30% for the testing set.

LDA model. Figure 4b. Shows the performance of the LDA model. The total number of best of PCs was as reported by the optimum accuracy rate achieved by CV. The optimum accuracy rate was 99.53% and 99.00% for the calibration and testing set, respectively, when PCs = 7. Table 2 displays the effect of preprocessing techniques applied. Detrend correction and first-order derivative (DC and 1st Der) were used to improve the accuracy of LDA in both the calibration and testing set, respectively.

LR model. Figure 4c. An illustration of the accuracy rate of the LR algorithm following CV. The LR as a linear regression attained an optimal discrimination rate after optimization when PC = 7. The best preprocessing method was the first-order derivative (1st Der) (Table 2). The best accuracy rate by the LR algorithm was 98.90% and 98.84% for the calibrations and testing set, respectively.

RF model. Figure 4d. A display of the accuracy of the RF algorithm. The total number of optimum PCs were as obtained in the excellent accuracy rate by CV. The optimum rate was 100% and 99.53% for the calibration and testing sets, respectively, at optimum PCs = 5. Table 2 displays the effect of preprocessing techniques applied. First-order derivative (1st Der) was used to improve the accuracy of RF in the calibration and testing set.

SVM model. Figure 4e. The accuracy rate of the SVM algorithm following the CV. The CV was carried out to verify the strength of the algorithm. Table 2 also shows the percentage enhancement by the preprocessing methods used. An accuracy rate for MC and MSC preprocessing techniques of 99.53% and 99.40% for calibration and testing set can be noted. As shown in Table 3, there are two sets of calibrated data (70%) and a test set (30%), which were used to evaluate the models and test their ability to classify.

#### 3.3.2. Evaluation of the Accuracy of the Discrimination Models

An assessment of the discrimination accuracy of the models was conducted using the well-known F-Score [48,49,50], which measures how good origin discrimination is in comparison with reference classification. It consists of the precision and recall values which are used in the extraction of information. The precision, Recall, and F-Score are defined as follows, and their values are given in Table 4.

(5) $$\mathrm{Precision}=\frac{\mathrm{True}\;\mathrm{positives}}{\mathrm{True}\;\mathrm{positives}+\mathrm{False}\;\mathrm{positives}}$$

(6) $$\mathrm{Recall}=\frac{\mathrm{True}\;\mathrm{positives}}{\mathrm{True}\;\mathrm{positives}+\mathrm{False}\;\mathrm{Negatives}}$$

(7) $$F-\mathrm{Score}=2\;\times \;\frac{\mathrm{Precision}\;\times \;\mathrm{Recall}}{\mathrm{Precision}+\;\mathrm{Recall}}$$

A Vis-NIR Spectroscopy was used to determine how millet varieties differ in how some incident radiation is reflected, transmitted, and absorbed. As presented in Figure 1, the emitted energy produced a series of stripes and a few peaks. The stripes are composed of overtones and a mixture of elemental vibrations, which are proportional to the natural properties of the samples. Despite their similarities when observed with the naked eye, many valuable and non-valuable properties exist. Hence, the need to apply ML algorithms to obtain useful information from each stripe. In addition, selecting the spectral area free of water is essential to reducing water absorption lines and noise. In this study, the spectral region of 400–2500 nm was chosen. The area has characteristics that may be used to distinguish millet varieties. Reflectance measurements were also made, and they revealed sixteen distinct classes (Figure 2), which illustrate the sixteen millet-producing regions. These differences result from physicochemical characteristics unique to each class.

The principal component analysis (PCA) converted the linear combinations of the original variables into new variables called principal components (PCs). It is due to differences in physical and chemical properties of the different classes. These PCs are orthogonal and positioned according to the interpretation of the variation. The first PC interpreted almost all the variations, followed by the second and third, etc. Generally, most of the variations were analyzed by the first few PCs [46]. Using the two PCs, the score biplots are employed to present the score space for sample groupings. The PCs score plots are shown in Figure 2. A distinct shape and color distinguish each variety for easier visualization. The PCs were normalized using their first three scores. It was found that the PC1, PC2, and PC3 explained 60%, 29%, and 5% of the total variance, respectively, whereas the first three PCs accounted for 94% of the total variance. The three peak PCs are particularly significant since they contain nearly the full spectral detail of the spectral regions in the NIR. It can be seen from Figure 3 that sixteen varieties could be distinguished with little overlap. There is a distinct separation between the varieties. Generally, millet samples from different geographical regions were separated. PCA analysis revealed that the chemical compositions of different varieties differed significantly. Even though the analysis produced a 3-dimensional cluster trend, the samples could not be discriminated.

**Figure 5:**
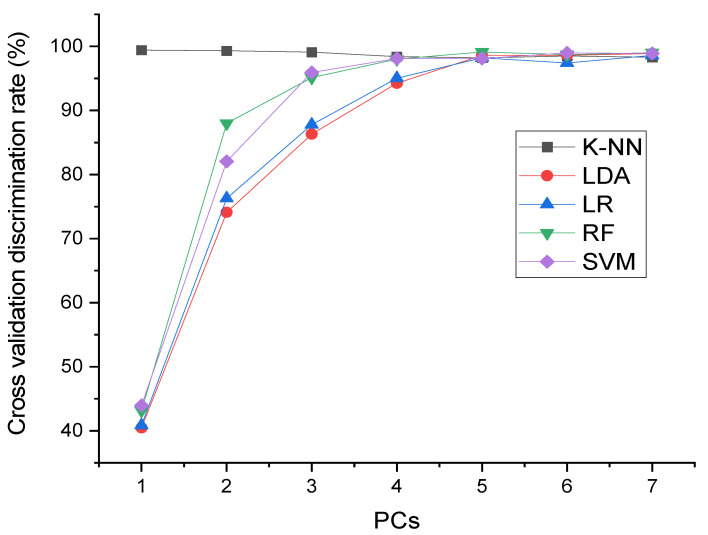
Cross-validation discrimination rates of different models at different PCs. K-NN: K-Nearest Neighbor; LDA: Linear Discriminant Analysis; LR: Logistic Regression; RF: Random Forest; SVM; Support Vector Machine.

Thus, five machine learning algorithms were used in this study, including K-NN, LDA, LRM, LR, RF, and SVM [28,34,36]. As illustrated in Figure 4 and Figure 5, CV was applied to verify the accuracy and capability of the algorithms. As can be observed, the two non-linear models (RF and SVM) performed slightly better than the three linear models (K-NN, LDA, and LR), which may be because non-linear models can perform well with high-dimensional data. In the case of k-NN, because it does not infer anything about features or data collection, it was speedy and moderately not fast, respectively, at calibration and prediction sets. It was determined that the algorithm obtained the k-nearest neighbors for every finding, which is not algorithmically straightforward. The LDA considered the entire data collection and evaluated the variability, which makes it susceptible to outliers. It may be that the good performance of LR is due to its ability to predict the target variable accurately when independent variables are uncorrelated with it and correlated with one another. As the total number of groupings and Decision Trees (DTs) increases, so does the efficiency of the RF algorithm. The RF method employed primary discrimination tests based on multiple arbitrarily generated subgroups, where the group with the greater number is regarded as the obtained discrimination outcome. The method employed a DTs bagging process and searched for the best feature among many indiscriminate features to improve the model’s predictability. Accordingly, the high accuracy achieved by SVM can be attributed to its flexibility and the ability to build generic models, even using just a few sample training data sets. The optimum separation hyperplane (OSH) is extrapolated from the data. OSH assumes that all classes are uniquely differentiated, which implies a model that can be adapted to a wide variety of situations. Finally, by considering the F-Score values, linear and non-linear algorithms (K-NN, LDA, LR, RF, and SVM) provided positive results. However, non-linear models appear to be slightly more accurate. Several factors may influence millet varieties depending upon their geographic origins, such as the condition and quality of the soil element in the different regions for ingestion by millet plants, and these characteristics generally depend on the soil’s cation exchange capacity pH and nutrient content. The differences can be influenced by crop maturity at harvest, artificial irrigation, fertilizer applications, and other agricultural practices in a particular region [51].

## 4. Conclusions

The discrimination capability of Vis-NIR Spectroscopy was demonstrated using machine-learning algorithms. Five algorithms were used, and millet samples were classified according to their geographical origins. For each model, cross-validation (CV) was conducted to optimize the classification accuracy, which was calculated from the highest classification rate. SVM and RF appeared to perform slightly better than the other linear models, with F-score values of 99.5%, LDA at 99.5%, K-NN at 99.1%, and LR at 98.8%. This study represents an essential contribution as few studies discuss several machine learning algorithms for millet geographic origin discrimination using NIRS due to the current trend in the EU for the establishment of Protected Geographical Indications (PGI) and Protected Designation of Origin (PDO). The investigation, however, observes some findings. There is a need for more robust origin models capable of better detecting regional and temporal variations in the future. Specifically, a large dataset representing wide variability (geographic origin, harvest period, and harvest year) should be analyzed.

## Figures and Tables

**Table 1:** Samples of varieties from different geographic regions.

Variety ID.	Variety Name	Producing Area	Number of Samples
S0	Qinxian yellow millet	Shanxi Province	30
S1	Mizhi oil millet	Shaanxi Province	30
S2	Huangjinmiao millet	Inner Mongolia	30
S3	Taohua yellow millet	Hebei Province	30
S4	Nandaobeimai millet	Liaoning Province	30
S5	Xirui yellow millet	Jilin Province	30
S6	Lucun millet	Shanxi Province	30
S7	Longshan millet	Shandong Province	30
S8	Qinzhou yellow millet	Shanxi Province	30
S9	Jinxiang Millet	Shandong Province	30
S10	Inner Mongolia yellow millet	Inner Mongolia	30
S11	Weizhou yellow millet	Hebei Province	30
S12	Fine yellow millet	Liaoning Province	30
S13	Organic red millet	Liaoning Province	30
S14	Black earth town organic millet	Heilongjiang Province	30
S15	Tian-di-Liang-ren organic yellow millet	Liaoning Province	30

**Table 2:** Effect of preprocessing techniques on the five different models.

Discrimination Rate (%)	Preprocessing	MSC	Detrend	MC	SNV	1st Der	2nd Der
K-NN	Calibration	100	99.80	99.90	100	100	100
	Testing	98.84	98.61	99.30	99.07	98.84	90.50
LDA	Calibration	99.30	99.53	99.53	99.76	99.53	89.68
	Testing	98.80	99.00	98.90	98.61	99.00	88.65
LR	Calibration	98.61	98.71	98.84	98.41	98.90	90.67
	Testing	98.37	98.61	98.80	98.37	98.84	88.88
RF	Calibration	100	100	100	100	100	100
	Testing	99.07	99.30	99.30	99.30	99.53	91.20
SVM	Calibration	99.30	99.30	99.53	99.20	99.53	95.53
	Testing	99.07	99.07	99.40	98.84	99.20	91.89

MSC: Multiplicative Scatter Correction; MC: Mean Centering; SNV: Standard Normal Variate; K-NN: K-Nearest Neighbor; LDA: Linear Discriminant Analysis; LR: Logistic Regression; RF: Random Forest; SVM; Support Vector Machine.

**Table 3:** The overall performance of the multivariate classification methods.

Models	Total Millet Samples		Discrimination Rate (%)	
	Calibration Set	Prediction Set	Calibration Set	Prediction Set
K-NN	1008	432	99.90	99.30
LDA	1008	432	99.53	99.00
LR	1008	432	98.90	98.84
RF	1008	432	100	99.53
SVM	1008	432	99.53	99.40

**Table 4:** Precision, Recall, and F-Score values.

Models	Precision	Recall	F-Score
K-NN	0.992	0.990	0.991
LDA	0.995	0.995	0.995
LR	0.988	0.988	0.988
RF	0.995	0.995	0.995
SVM	0.995	0.995	0.995

## Data Availability

The data presented in this study are available on request from the corresponding author. The data are not publicly available due to privacy.
